# Do Online Mental Health Services Improve Help-Seeking for Young People? A Systematic Review

**DOI:** 10.2196/jmir.3103

**Published:** 2014-03-04

**Authors:** Sylvia Deidre Kauer, Cheryl Mangan, Lena Sanci

**Affiliations:** ^1^Department of General PracticeUniversity of MelbourneCarltonAustralia; ^2^The Inspire FoundationSydneyAustralia

**Keywords:** adolescent, young adult, Internet, medical informatics, mental health, mental disorders, systematic review, information seeking behavior

## Abstract

**Background:**

Young people regularly use online services to seek help and look for information about mental health problems. Yet little is known about the effects that online services have on mental health and whether these services facilitate help-seeking in young people.

**Objective:**

This systematic review investigates the effectiveness of online services in facilitating mental health help-seeking in young people.

**Methods:**

Using the Preferred Reporting Items for Systematic Reviews and Meta-Analyses (PRISMA) guidelines, literature searches were conducted in PubMed, PsycINFO, and the Cochrane library. Out of 608 publications identified, 18 studies fulfilled the inclusion criteria of investigating online mental health services and help-seeking in young people aged 14-25 years.

**Results:**

Two qualitative, 12 cross-sectional, one quasi-experimental, and three randomized controlled trials (RCTs) were reviewed. There was no change in help-seeking behavior found in the RCTs, while the quasi-experimental study found a slight but significant increase in help-seeking. The cross-sectional studies reported that online services facilitated seeking help from a professional source for an average of 35% of users. The majority of the studies included small sample sizes and a high proportion of young women. Help-seeking was often a secondary outcome, with only 22% (4/18) of studies using adequate measures of help-seeking. The majority of studies identified in this review were of low quality and likely to be biased. Across all studies, young people regularly used and were generally satisfied with online mental health resources. Facilitators and barriers to help-seeking were also identified.

**Conclusions:**

Few studies examine the effects of online services on mental health help-seeking. Further research is needed to determine whether online mental health services effectively facilitate help-seeking for young people.

## Introduction

Mental health problems affect adolescents more than any other age group [1]. Help-seeking is an important first step in improving mental health and accessing appropriate avenues of care [2]. Defined as “the process of actively seeking out and utilizing social relationships, either formal or informal, to help with personal problems” (p. 8) [2], help-seeking is a complex process involving awareness and appraisal of the problem, the ability to express the problem and need for support, relying on accessible and available sources of help, and a willingness to seek out and disclose relevant information [2]. In the last decade, the Internet has become a predominant source of health information [3,4] particularly for young people [5,6]. Various online services are readily available for young people including self-directed, low intensity Web-based mental health support (eg, ReachOut), national online counseling services (eg, eheadspace), repositories for information and resources concerning mental health (eg, Somazone), and structured self-directed online therapy (eg, MoodGym [7]). Online mental health services may conceivably assist in all elements of the help-seeking process.

Despite large investments in mental health reforms, face-to-face services are unable to support the large number of young people experiencing mental health problems [8,9]. In addition, existing face-to-face services pose significant barriers for young people [10-16] largely due to access, availability, and high costs of these services [17], as well as the reluctance of young people to seek professional help due to stigma and embarrassment [18-20]. Wilson and colleagues [21] identified that young people have a strong desire for autonomy, believing they should solve problems for themselves. In terms of these barriers, Internet services have several advantages: no geographical boundaries, services are generally free to the user, and the Internet is largely anonymous and private, which is likely to reduce the stigma and embarrassment associated with seeking help [22]. The interactive nature of the Internet allows for the provision of online therapies in various forms such as games or eLearning websites [23,24]. Online mental health services can provide interactive solutions to engage young people in a self-directed and anonymous way, thus assisting and supporting overburdened face-to-face services. Understanding young people’s readiness for care is a key factor in supporting young people in reaching services appropriate to their needs [16]. Web-based technology can deliver stepped care services [25], providing non-intrusive treatments for those with mild problems and increasing with intensity as required.

It is important to ensure that young people are aware of local face-to-face services as well as online options, particularly for young people with severe mental health problems who require intensive treatment or medication. Online directories can facilitate pathways to face-to-face services as well as online care [24,26], allowing young people who need intensive services to readily access them while also supporting the large number of young people with mild or moderate mental health concerns.

Routinely, investigations into health and mental health websites has involved evaluation of (1) the quality of the information [3,27-30], (2) the scope and reach of the website [31-33], and (3) consumer satisfaction [34,35]. There is also considerable research demonstrating that structured online therapy programs (eg, MoodGym) effectively improve mental health outcomes [36-40] and that mobile self-monitoring is a useful tool [41-43]. Online mental health websites have also been shown to increase the use of services for adults [44]; however, the effect of online information services and other regularly used unstructured websites on help-seeking in young people is rarely explored [45].

As improving help-seeking is integral to accessing care and improving mental health, this systematic review investigates the effectiveness of current online mental health services in facilitating the help-seeking process in young people. The aims of this review are to explore past literature that investigate whether online mental health services facilitate the help-seeking process in young people, specifically focusing on help-seeking behaviors, the barriers and facilitators influencing online help-seeking, and the experiences of young people who use these services.

## Methods

### Literature Search

This review was conducted in accordance with PRISMA guidelines [46] and registered on PROSPERO (Prospero Registration Number: CRD42013003904)[47]. Peer-reviewed English citations up to February 27, 2013, on the databases PsycINFO, PubMed, and the Cochrane Library, were searched using search terms representing three concepts: Web-based technology, mental health, and help-seeking (details described in Multimedia Appendix 1). All controlled and uncontrolled studies were eligible for inclusion including qualitative studies. The reference lists of all relevant studies, reviews, and meta-analyses identified in this search were also manually searched for inclusion into the review.

### Selection of Studies

The first author examined all titles and abstracts extracted for relevance and read the full text for any potentially eligible article. Table 1 describes the exclusion criteria. The second author confirmed that all selected articles were eligible for inclusion.

**Table 1:** Exclusion criteria.

Criteria	Description
Over 25 years of age	Depending on how age was described in the study, studies were excluded when over 25% of participants in the study were not young people between the ages of 14 and 25 years (per Gulliver [13]); the average age of participants was >30 years; or no age was mentioned and the article referred to adults.
Not mental health	The outcome addressed was a health outcome other than mental health (eg, diabetes, heart disease).
Not Web-based	There was no technological component discussed such as online mental health services.
Unrelated technology	The online program did not specifically explore mental health (eg, the effects of popular computer games or social networking sites on mental health outcomes). Online forums were excluded except when they were specifically dealing with mental health issues (eg, forums specifically for young people with depression).
Electronic medical records	The program was an electronic system to keep medical records.
No evaluation	The paper described the program but did not evaluate help-seeking behavior, attitudes, or intentions.
Not a study	The paper was not a study (ie, a review paper or discussion piece). Any relevant studies in reference lists were included.
Third-party program	The study focused on a third party seeking help for the young person (eg, parent or teacher).

### Coding of Studies

The first author extracted data from the studies using the form described in Table 2, which was confirmed by the second author. Risk of bias within each study was assessed using a version (Multimedia Appendix 2) of the Quality Rating Scale (QRS) [48] adapted to include qualitative and uncontrolled studies. The first two authors used the QRS independently. Interrater reliability was assessed by Cohen’s absolute weighted kappa statistic in Stata Version 12.0. Weighted kappa allows for different levels of agreement in ordered data, and the absolute function allows for all numbers including those unassigned by either rater.

The country, research group, and year published were also examined to determine whether there were biases across studies in terms of the country of research, research group, or years in which the research was conducted. All studies were included irrespective of their design, quality, and biases. As suggested by Linde and colleagues [49], this review included uncontrolled trials and qualitative studies to obtain an overview of the topic and inform future research. No statistical analyses were conducted due to the heterogeneity of study designs permitted in the inclusion criteria. Instead, a broad qualitative overview of the data was conducted and statistical analyses from the studies were reported.

**Table 2:** The pre-determined form used to code the selected studies.

Reference	Authors’ names, research group (including the department, organization, and country), and the year published
Sample	Number of participants, age of participants (either mean and standard deviation, range, or percentage of young people), percentage of female participants, population targeted (ie, mental health status or risk profile)
Study design	Qualitative or quantitative (cross-sectional, quasi-experimental, longitudinal, RCT)
Program	Type of online mental health services examined (eg, information website, discussion forums, screening tool, online therapy)
Outcome	Outcomes studied (ie, mental health or help-seeking outcomes, user satisfaction, economic evaluation), the measures used to assess the outcome, and summary statistics reported

## Results

### Summary

**Figure 1:**
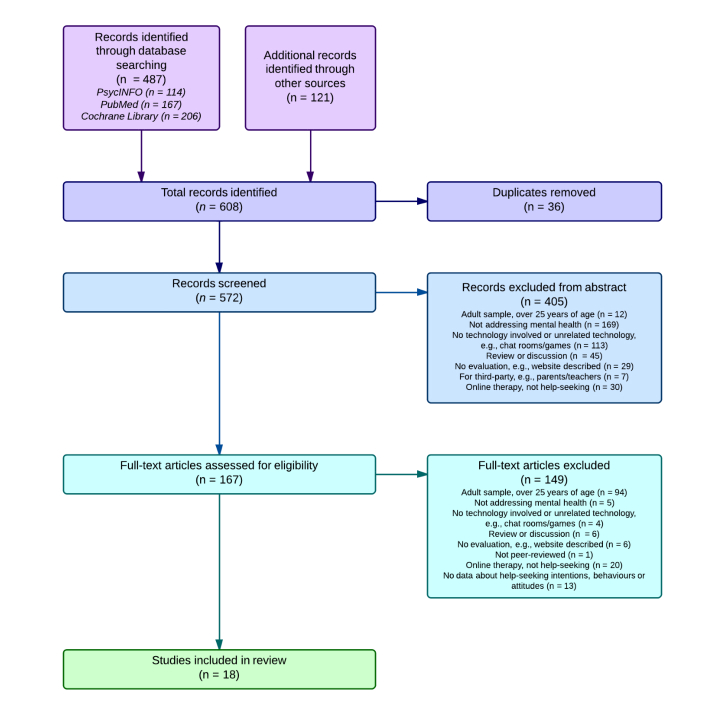
PRISMA flow diagram for inclusion into the review.

A total of 487 articles were identified through the literature search. All relevant review and research papers [37,41,50-70] were then manually searched, which uncovered a further 121 potential studies. Of these 608 papers, 405 were excluded based on their abstracts and a further 149 excluded after the full article was examined, leaving a total of 18 studies to review. Figure 1 depicts the PRISMA flow diagram for inclusion.

### Study Characteristics

Eighteen publications met the inclusion criteria including two qualitative [51,55], 12 cross-sectional [53,56,60,63,64,71-77], one quasi-experimental [68], and three randomized controlled trials (RCTs) [52,78,79]. The sample sizes ranged from 9 to 2700: median 420, mean 762.3 (SD 838.10). There were a high proportion of females in the studies, ranging from 50% to 80%: median 68%, mean 67.1% (SD 9.73%). The characteristics of each study are presented in Table 3.

The majority of participants were students at a university [55,56,60,63,74,76] or high school [73]. Other studies involved users of online services [53,68,72,75,77]. Within these settings, 10 studies targeted young people with mild to moderate mental health problems [52,53,55,63,64,68,72,75-77], one study focused on young athletes at risk of mental health problems [79], and seven studies involved all young people regardless of mental health status [51,56,60,71,73,74,78].

The types of programs investigated were varied as outlined in Table 3. Most commonly, the use of several programs and websites [56,60,71,73,74,76] were investigated, such as online information sites, chat groups, self-directed online therapy, and forums. Other studies investigated a specific information site [53,64,77-79], a self-directed online therapy [51,52,55], a discussion forum about mental health problems [72,75], a Web-based mental health educational game [68], and a screening tool [63].

**Figure 2:**
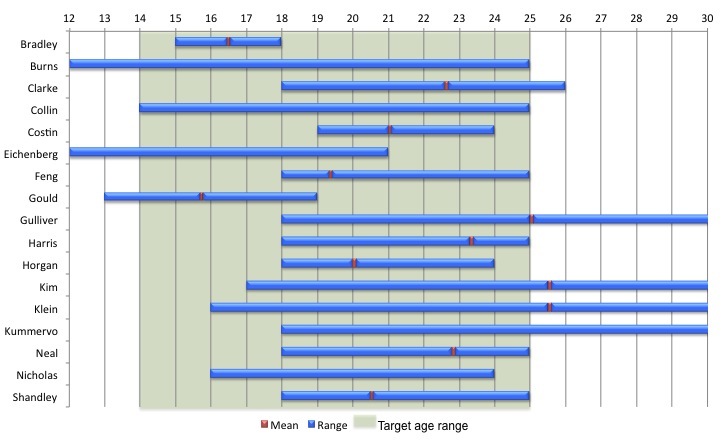
Age range and means for each study, with target age range indicated (mean age was not reported for some studies; end age range exceeded 30 years for the Klein study [70 years]).

Figure 2 shows the age ranges and means for each study. Most studies (11/18) were inclusive of the target age range; 55% (10/18) of studies focused on participants who were 18 years of age or older. The mean age of participants for the studies ranged from 16.5-26.2 years of age.

**Table 3:** Study type, target group, service evaluated, sample, study design, and findings related to help-seeking of the included studies.

Study		Target group / online service	Sample / design^a^	Help-seeking findings
**Discussion forums**				
	Eichenberg [72]	Message board posts were examined from adolescents who used the forum “Selbstmord” and users completed a survey	N=164 50% female CS	38/164 (23%) answered “yes” to the question “did you use the forum in order to obtain information about professional help?”
	Kummervold et al [75]	Users of the forums Doktor Online & SOL Helse completed a survey	N=492 78% female CS	19/165 (6%) answered “yes” to the question “did participating in the forum increase your use of traditional services?”
**Web-based mental health educational game**				
	Shandley et al [68]	Young Australians who registered to play ReachOut Central	N=266 66% female QUASI	A significant increase in willingness to seek help was found from pre-test (male mean 4.01 [SD 2.98]; female mean 4.17 [SD 2.69]) to post test (male mean 4.39 [SD 3.13], female mean 5.04 [SD 2.99]); *F*_1,264_=18.04, *P*<.001.
**Internet-based self-help program**				
	Bradley et al [51]	Pilot study with 15-18 year olds with no previous mental illness scoring below severe on the Depression, Anxiety and Stress Scale completed the Feeling Better Program	N=13 69% female QUAL	Participants qualitatively expressed that they thought the online mental health service improved accessibility to help
	Clarke et al [52]	Depressed and non-depressed 18-24 year olds were recruited and randomized into an online self-help program or treatment as usual	N=160 80% female RCT	The chi-square test revealed no significant difference for mental health visits between the intervention (mean 1.3 [SD 3.1]) and the control group (mean 0.9 [SD 2.7]).
	Davis-McCabe & Winthrop [55]	Pilot study of university students who used the Computer Aided Lifestyle Management program	N=9^a^ QUAL	Participants “saw the program as a useful tool that aids further self-help” (p. 51)
	Gulliver et al [79]	Elite athletes were randomized into three versions of an online self-help program: mental health literacy and de-stigmatism, a feedback condition about symptoms, or a minimal content condition involving a list of help-seeking resources or a waitlist control group	N=59 72% female RCT	No difference in help-seeking behavior was found between any of the intervention groups and the control group over time (mental health literacy/destigmatization OR 57.38, 95% CI 0.85-3868.09, *P*=.06; feedback OR 5.15, 95% CI 0.04-637.04, *P*=.51; help-seeking list OR 57.38, 95% CI 0.15-1263.93, *P*=.25).
	Collin et al [53]	Young people who used ReachOut.com completed a survey	N=2291 76% female CS	546/1552 (35.2%) of participants said “yes” to “Did ReachOut.com help you ask a professional for help?”
	Costin et al [78]	Random representative sample of 19-24 year olds recruited through a postal mail-out randomized to (1) receiving brief, (2) advanced or (3) non-related depression health e-cards	N=348 75% female RCT	No increase was found in help-seeking on the AHSQ for formal (OR 1.17, χ^2^_1_=0.14, *P*=.70) or informal (OR 0.86, χ^2^_1_=0.18, *P*=.67) sources.
	Klein et al [64]	Drug and alcohol users who accessed at least 1 of 14 drug and alcohol websites completed a survey	N=1214 66% female CS	336/994 (38.8%) of participants indicated a preference for websites with email support from a therapist
	Nicholas [77]	Young people who used ReachOut.com completed a survey	N=1016^b^ CS	386/1016 (38%) of participants said yes to “Are you more likely to talk to a mental health professional after visiting ReachOut.com?”
**Online screening tool**				
	Kim et al [63]	University students used a Web-based self-screening and referral system and completed a survey	N=2700 68% female CS	43/57 (75.4%) of participants said “yes” to “Did the screening tool help you make your decision to see a mental health care professional?”
**Multiple online services**				
	Burns et al [71]	Random representative sample of 12-15 year olds via telephone were asked their opinions about online mental health services	N=2000 50% female CS	70/227 (30.8%) of participants said “yes” to “Have you ever used the Internet to find information for a mental health, alcohol, or other substance use problem?”
	Feng & Campbell [56]	University students were asked their opinions about online mental health services	N=176 68% female CS	77/176 (44%) of participants said “yes” to “Have you used the Internet in the past to learn about personal feelings, anxiety, sadness, or confusion?”
	Gould et al [73]	High school students were asked their opinions about online mental health services	N=519 50% female CS	94/519 (18%) of participants said “yes” to “In the past 12 months, did you use the Internet to seek help when you felt very upset, sad, stressed or angry?”
	Harris et al [60]	University students were asked about the online mental health services used to seek help for suicidal ideation	N=64 75% female CS	24/64 (37.5%) of participants said “yes” to “How likely would you be to use a help-site?”
	Horgan & Sweeney [74]	University students completed a survey about their opinions about online mental health services	N=922 62% female CS	267/867 (30.8%) of participants said “yes” to “Have you used the Internet for mental health information?”
	Neal et al [76]	University students were asked their opinions about online tools	N=1308 68% female CS	698/1308 (53%) of participants said “yes” to “Have you used the Internet in the past for information on mental health when sad, anxious or confused?”

^a^CS=cross-sectional; QUAL=qualitative study; QUASI=quasi-experimental.

^b^Percentage of females not provided.

### Primary Outcomes

#### Overview

Help-seeking behavior or service utilization was the primary outcome in four studies [53,63,78,79]. The primary outcomes for the remaining studies were the characteristics of young people who sought help online [56,60,72-77], mental health [52,55,68], and process evaluation of online services [51,64,71]. These are summarized below.

#### Help-Seeking

Studies were included in this review only if they explored help-seeking, yet only four studies included help-seeking as their primary aim, two of which were RCTs [52,78,79] and two were cross-sectional studies [53,63]. Costin et al [78] hypothesized that young people who received the intervention (depression information eHealth cards) would be more likely to seek professional health care than those who received the attention-control condition (non-depression information eHealth card). This hypothesis was not supported as no difference between the groups was found. Gulliver et al [79] investigated the effects of three conditions of an Internet-based self-help program on help-seeking attitudes, intentions, and behavior and found no difference in help-seeking between groups. Kim et al [63] conducted a cross-sectional study to examine the ability of an online screening tool to help students assess their mental health and encourage them to seek treatment. The majority of students (75.4%) indicated that screening enhanced their decision to see a professional. Collin et al [53] examined young people’s engagement with an online mental health service and how the service facilitated help-seeking in a cross-sectional study and found that a minority of users (35.2%) thought the service helped them ask a professional for help.

Overall, no increase in help-seeking or health utilization was found in the three RCTs for the intervention groups compared to control groups (these statistics are found in Table 3). The lack of change in these studies may have been due to small sample sizes, an active comparison group [52], mild mental health problems rather than severe symptoms [79], or the fact that participants had previously sought help or were currently in treatment [78]. In the quasi-experimental study [68], participants rated on a 10-point scale: “if you felt sad, down, or miserable for >2 weeks, how likely is it that you would get help from a counsellor, psychologist, or psychiatrist?” There was a statistically significant increase in willingness to seek help (as listed in Table 3); however, the post-test mean was 5.01 for females and 4.39 for males, suggesting a low to medium score on the 10-point scale.

Of the cross-sectional studies, two general types of questions were asked: questions about Internet help-seeking behaviors (eg, “have you ever used the Internet for mental health information?”) [56,60,71,73,74,76] and questions about the use of the Internet to seek professional help (eg, “did the service help you make your decision about seeing a professional?”) [53,63,72,75,77]. The mean percentage of young people answering “yes” to these questions across studies was low, 38.4% (range 18% to 53%) and 34.9% (range 6% to 75.8%) respectively.

#### Descriptive Characteristics

Feng et al [56] explored the relationship between personality types and online help-seeking and found no association, all participants were equally likely to engage in online help-seeking. Eichenberg [72] assessed whether online message forums for people to discuss suicidality were harmful and found that users primarily used the site for constructive reasons. Three type of users participated in the suicide forums: those with destructive motives who participated for constructive reasons (21%, 23/108), those without a clear motivation for visiting (31%, 33/108), and those with constructive motives (48%, 52/108). Gould et al [73] investigated demographic characteristics, hopelessness, and functional impairment with the use of online services and found that at-risk young people were using online help-seeking in combination with other services, rather than substituting online services for other resources. Horgan and Sweeney [74] asked young people about their views and usage of Internet help-seeking and found that 267 out of 867 (30.8%) used the Internet to search for mental health information, 593 out of 872 (68%) indicated they would use the Internet if they needed to, and 689 out of 868 (79.4%) said they would prefer face-to-face support. Neal et al [76] also explored young people’s opinions of online mental health help and how well the program engaged young adults’ attention. Most young adults used Google to find mental health information and were unaware of potential websites that may assist them. Nicholas [77] investigated the perceived effects of the ReachOut site on its users and found that 37% (376/1016) of users visited the site more than once a week, 83% (832/1016) reported learning more about mental health issues, and 77% (872/1016) reported learning where to get help. Harris et al [60] investigated young people’s intentions to use online sources and found that previous phone helpline usage, a suppressive problem-solving approach, and not seeking face-to-face professional services were associated with an increase in seeking help online (*R* ^2^=.32, *F*_3,60_=9.44, *P*<.001). Kummervold and colleagues [75] examined who used mental health forums, why they used them, and the implications of forum use and found that women were the predominant users of forums (78%, 384/492), 75% (306/408) found it easier to discuss problems online compared to face-to-face, and 62% (195/317) used the forums as a supplement to mental health services. Professionals were welcome to participate and pseudonyms were important to 68% (334/492) of users.

#### Mental Health

The qualitative study by Davis-McCabe and Winthrop [55] investigated the potential of an online self-help program at a university and found that students reported positive changes and experiences from the program. Clarke and colleagues [52] compared an online program with treatment-as-usual and found that young adults with depressive symptoms had a modest yet significant reduction in symptoms post test (N=160, *d*=0.20, 95% CI 0.00-0.50) with a moderate effect for women (n=128, *d*=0.42, 95% CI 0.09-0.77). Shandley et al [68] evaluated the benefits of an online game designed to help young people with mental health problems and found slight but significant improvements in problem-solving (post test: *F*_1,264_=4.42, *P*=.04; follow-up: *F*_1,264_=4.92, *P*=.03), seeking support (follow-up: *F*_1,264_=7.70, *P*=.01), avoidance (post test: *F*_1,264_=4.10, *P*=.04; follow-up: *F*_1,264_=3.94, *P*=.04), psychological distress (post test: *F*_1,264_=11.89, *P*<.001; follow-up: *F*_1,264_=9.04, *P*<.001), resilience (post test: *F*_1,264_=5.87, *P*=.02; follow-up: *F*_1,264_=10.86, *P*<.001), and satisfaction with life (post test: *F*_1,264_=4.68, *P*=.03; follow-up: *F*_1,264_=4.70, *P*=.03).

#### Process Evaluation

Burns [71] explored young people’s Internet use for mental health resources and found that the Internet was used by 76.9% (1464/1905) of young people to connect with peers and by 38.8% (735/1894) of young people to seek information about mental health. Bradley et al [51] investigated what young people wanted in an Internet program for psychological distress and using thematic analysis found that usefulness, credibility, privacy, convenience, and accessibility were important to young people as well as being aware of the program and having the motivation to use the program. Klein et al [64] explored individual preferences for content and functionality on alcohol and other drug websites and found that interactive functionality (being asked a question: χ^2^_10=_36.1, *P*<.001; consumer hub: χ^2^_10=_34.6, *P*<.001) and social networking features (chat room access: χ^2^_10_ =28.9, *P*<.001; blogging feature: χ^2^_10=_53.3, *P*<.001) were valued by younger adults compared to older adults.

### Barriers and Facilitators to Online Help-Seeking

A range of barriers and facilitators of help-seeking were mentioned in the articles, although these were rarely measured empirically. Barriers to help-seeking included lack of awareness [51,56,76], being male [68,71,73], a preference for face-to-face services [73,74], lack of motivation [51], uncertainty about confidentiality [74], and unfavorable content [64,76]. Facilitators to help-seeking included accessibility to online resources [51,53,55,71,74], the ease of sharing personal information compared to face-to-face services [74-76], anonymity [51,71,74,75], trust and credibility [53], reduction of stigma [55,68,79], high distress [55,73], and an increase in mental health literacy [53,68,74-79]. Table 4 describes these facilitators and barriers in detail.

**Table 4:** Barriers and facilitators of online mental health resources.

Theme (n)		Findings
**Barriers to online mental health services**		
	Lack of awareness (3)	Young people reported a lack of awareness of the online mental health services available [51,56,76].
		“Many participants commented that they would not have thought to look for online distress-management programs” (Bradley et al [51], p. 30).
		Young people most commonly used text-based search engines and information sites and were either not aware of other online mental health resources [56,76] or did not believe that they would help [76].
	Online mental health services are not everyone’s preferred source (2)	Young people were more likely to seek help from informal sources such as friends and parents when compared to Internet resources [73].
		Some young people preferred face-to-face services to Internet services [74].
	Males don’t seek help online (3)	Young men were less likely to seek help using online mental health services than young women (χ^2^=8.6, *P*<.01) [71].
		After playing ReachOut Central [68], young women were marginally more willing to seek help than young men.
		Gould et al [73] showed no gender differences in frequency of online help-seeking behavior.
	Lack of motivation (1)	Some distressed young people would not be motivated to seek help on or offline. “You have to want to help yourself” [51] p 30.
	Not confidential / impersonal (1)	Online mental health services were reported by some participants [74] as unreliable (15.1%), untrustworthy (5%), lacking in privacy (2.5%), impersonal (7.5%), and as providing insufficient support (3.9%).
	Content unfavorable (2)	Information websites can to be too technical to understand [76], forums contain inaccurate information, with people complaining and unhelpful [76], and finding the desired information on alcohol websites can be difficult [64].
**Facilitators to online help-seeking**		
	Accessibility of online mental health resources (5)	67.3% of users agreed that ReachOut was “there when they need it” [53]. Horgan and Sweeney [74] reported that participants used the Internet for mental health support because of the vast amounts of information valuable online (21.8%), the Internet is easily accessed 24 hours (10.1%), it is easy to find information (11%), fast (8.3%), cheap (2.7%), convenient (1.7%), and a good place to start and find out where to go for further assistance (4.7%). One of the useful features of online mental health services is accessibility as they are available when required in crisis situations when other support cannot be reached, particularly flexible programs that allow users to come and go [55].
		In the qualitative study by Bradley et al [51], participants expressed that they thought online mental health services improved accessibility to help; “anyone, anywhere, anytime” (p. 29).
		Late night Internet usage is another benefit to online mental health services [71], with Internet use after 11 p.m. the only predictor for young men to use the Internet as a mental health resource (OR 2.1, CI 1.1-4.3).
	Anonymity (4)	In one study [74], 22.3% of participants preferred online mental health services to face-to-face services because they are anonymous, private, and confidential.
		In another study [75], 64% of participants valued the anonymity that the program allowed and indicated that they would not have used the service if they had to give their real name.
		Bradley et al [51] reported that participants felt that online mental health services were the most private way to seek help; however, Burns et al [71] found that young people did not consider anonymity helpful.
	Trust/credibility (1)	Only one study [53] examined how trustworthy young people found online mental health services to be and found that the majority of visitors agreed that ReachOut.com was relevant, credible, and trustworthy (74.3%). Participants preferred online mental health services to face-to-face services because they believed they would not be judged (7.3%).
	Reduction of stigma (3)	The results from Gulliver et al [79] demonstrated a significant interaction between the type of program and time in depression stigma (*F*_667.51_=3.99, *P=*.002) and anxiety stigma (*F*_662.22_=3.20, *P=*.008). Further investigation showed that the mental health literacy/destigmatization condition had a greater decrease in stigma than the other two conditions. There was no difference in stigma from pre-test to follow-up in the evaluation of ReachOut Central [68].
		One study [55] qualitatively found that online services were associated with less stigma than face-to-face services.
	Sharing personal information (5)	Two benefits of discussion forums were mentioned: (1) reading and posting on discussion forums demonstrated to young people that there are other people going through similar problems and that they were not alone [76], and (2) 75% of young people said it was easier to discuss personal problems using online mental health services than face-to-face services and that they would discuss things they would not offline [75].
		Some of the reasons for using the Internet for mental health support in Horgan and Sweeney’s study [74] included ease of communicating with other young people (3.7%), more inclined to open up on the Internet (1.7%), believed it would be easier to express themselves online (1.1%), and discussing personal problems online is less embarrassing than talking to a professional, friend, or family member (2.9%).
		Interestingly, two studies indicate an association between distress and help-seeking online [55,73].
	High distress (2)	Participants in Davis-McCabe and Winthrop’s study [55] reported that they were more likely to use online mental health services when distressed.
		Similarly, young people who were functionally impaired (ie, scoring >16 on the CIS) were more likely than unimpaired to seek information online (34% versus 20.6%; χ^2^=7.4, *P*<.01) as were young people scoring nine or above on the Beck Hopelessness Scale when compared to those with low scores (16.1 versus 9.1%; χ^2^=3.8, *P*>.05) [73].
	Increase of mental health literacy (8)	Eight studies explored mental health literacy. Participation in the mental health literacy/destigmatization condition in Gulliver et al’s study [79] demonstrated a greater increase in depression literacy; *F*_669.41_=2.47, *P*=.03) and anxiety literacy; *F*_667.51_= 3.99, *P=*.002, compared to the other conditions.
		Users reported that ReachOut.com contributed to their knowledge about mental health issues: 43.3% learned skills, knowledge, and confidence to seek help if they needed it [53], 57% gained an understanding about mental health issues [53], and 77% said they learned where to get help [77].
		Neal et al [76] reported that several participants indicated in their qualitative responses that information websites helped learn about their feelings. Those who had previously sought online help rated all online mental health services higher than those who had not previously sought help [76]. A small proportion of participants (4.7%) said that they used online mental health services because they believed it would be a good place to get initial information about mental health [74].
		Forum users believed the forum increased their knowledge and understanding of mental problems, health care services, their rights, and what they could expect from health services [75]. In addition, being part of the forum made them feel more pro-active, prepared, and goal-oriented with seeking help [75].
		ReachOut Central, however, was not associated with an increase in mental health literacy for males or females [68]. No difference was found in help-seeking knowledge, ability to recognize depression, or intentions to seek help between different information eHealth cards, although an increase in beliefs about efficacy of formal help-seeking from pre-test to post test [78] was found.

### User Experience

Young people’s experiences with online services were investigated in 50% (9/18) of the studies. Generally, participants were asked to rate how helpful the service was [56,63,71,73,76,78], how easy it was to use [63,68], whether they would use it again [63,68], whether they would recommend it to others [53,68,71,73], or if they were satisfied with the service [63,71,73]. There was high variability in the measures used, and therefore the results are difficult to compare between studies. All of these studies used from one to six questions, which were constructed by the authors. No standard measures were used. User experience is summarized in Table 5.

Overall, experiences of the online services were positive. Of the nine studies that evaluated these experiences, 90% of participants were satisfied with the service, 86% would continue to use the service or use it again in the future, and 72% would recommend it to a friend. However, approximately half of participants received the information they were looking for, and only 65% found the programs helpful.

**Table 5:** User experience of online services.

Theme (n)	Overall percentage (n/N)	Individual studies percentage (n/N)
Somewhat to very helpful (6)	65.50% (1156/1765)	85.96% (98/114) [78]
		78.11% (571/731) [71]
		55.35% (383/692) [76]
		50.88% (29/57) [63]
		45.74% (43/94) [73]
		41.56% (32/77) [56]
Somewhat to very satisfied (3)	90.35% (796/881)	93.57% (684/731) [71]
		71.28% (67/94) [73]
		80.36% (45/56) [63]
Would recommend to a friend (4)	71.94% (1838/2555)	88.20% (157/178) [68]
		84.95% (621/731) [71]
		65.98% (1024/1552) [53]
		38.90% (36/94) [73]
Received the information they wanted (2)	52.18% (1595/3057)	49.11% (359/731) [71]
		50.68% (596/1176) and 55.65% (640/1150) found it within 5 to 15 minutes [64]
Ease of use (2)	91.91% (216/235)	98.25% (56/57) [63]
		89.89% (160/178) [68]
Would continue to use (2)	86.38% (279/323)	89.29% (50/56) [63]
		86.09% (229/266) [68]

### Assessment of Bias

The adapted QRS ranged from 6 to 37 for both raters and had similar means (Rater 1 mean 19.5 [SD 7.45]; Rater 2 mean 20.2 [SD 8.66]). The qualitative and cross-sectional studies scored low, between 6 and 24 for both raters. The quasi-experimental study [68] scored 20 by rater 1 and 29 by rater 2, and the RCTs scored high from 31 to 37 by both raters. Interrater agreement was higher (89.8%) than the expected agreement (76.1%) with an acceptable kappa score (κ=.57, CI 0.32-0.82, *P*<.001). All studies were published between 2002 and 2012 (median 2010), with 15 studies occurring in the last 5 years (2009-2012). Seven studies [52,53,55,63,77-79] developed the intervention that they were evaluating, which may have led to a bias towards more favorable reporting.

There was a bias towards affiliations with four organizations, with nine studies conducted within Australia compared to three in the United States, two in Canada, and one each in Germany, Ireland, Norway, and United Kingdom. Three studies were from the Inspire Foundation, Sydney [53,71,77], two from the National eTherapy Centre at Swinburne University in Melbourne [64,68], two from the Centre for Mental Health Research at Australian National University in Canberra [78,79], and two from the Faculty of Health Sciences at the University of Sydney [56,76]. The remaining nine were not affiliated with the other studies included in this review. Table 6 lists the study, research group, year of publication, country where the research was conducted, and QRS.

**Table 6:** Assessment of bias for each study.

Study	Research group	Year published	Quality^a^
Bradley et al [51]	IWK Health Centre, Canada	2012	11.5
Burns et al [71]	Inspire Foundation Australia	2010	19
Clarke et al [52]	Kaiser Permanente Center for Health Research, United States	2009	34
Collin et al [53]	Inspire Foundation Australia	2011	19.5
Costin et al [78]	Centre for Mental Health Research, Australian National University, Australia	2009	34
Davis-McCabe & Winthrop [55]	Counselling Psychology, Teeside University, United Kingdom	2010	6
Eichenberg [72]	Institute of Clinical Psychology and Psychotherapy, University of Cologne, Germany	2008	16
Feng & Campbell [56]	Department of Health Science, University of Sydney, Australia	2011	21.5
Gould et al [73]	Columbia University, United States	2002	19.5
Gulliver et al [79]	Centre for Mental Health Research, Australian National University, Australia	2012	34
Harris et al [60]	School of Psychology, University of Queensland, Australia	2009	17.5
Horgan & Sweeney [74]	Brookfield Health Science Complex, University College Cork, Ireland	2010	20
Kim et al [63]	Department of BioEngineering, University of Washington, United States	2011	19
Klein et al [64]	National eTherapy Centre, Swinburne University, Australia	2010	17
Kummervold et al [75]	Norwegian Centre for Telemedicine, University Hospital of North Norway	2002	16
Neal et al [76]	Faculty to Information and Media Studies, University of Western Ontario, Canada	2011	17
Nicholas [77]	Inspire Foundation Australia	2010	11
Shandley et al [68]	National eTherapy Centre, Swinburne University, Australia	2010	24.5

^a^Average score on the Quality Rating Scale between the two raters.

### Limitations of the Studies

The majority of studies included a comprehensive list of limitations: small sample size [51,52,55,60,68,78,79], resulting in insufficient power to detect a change [52,68,78,79]; a self-selected sample, not representative and possibly biased [56,60,63,68,73,77-79]; majority of participants were female [51,56,60,64,68,79]; a lack of longitudinal tracking in the study [53,71,77]; only one online mental health service was investigated [51]; only one behavior change theory was investigated [51]; limited outcome measures [52,60,64,73]; insufficient length of follow-up time points [78]; no quantitative analysis [55]; non-validated and possibly biased measures [56,64,74,77]; and lack of qualitative information to provide depth about attitudes [77].

## Discussion

### Principal Findings

There is a plethora of online services available with the aim of facilitating help-seeking for young people with mental health problems [80], yet only 18 studies were identified in this review that evaluated whether these services increased help-seeking in young people. Overall, these studies did not indicate that online services facilitate mental health help-seeking in young people. No change in help-seeking was found in the three RCTs [52,78,79]. The quasi-experimental study found a slight but significant increase in help-seeking [52,68,78,79]. The cross-sectional studies found that only 35% of participants indicated the services helped them seek help from a professional. Only 52% of participant also reported that they received the information they wanted, and only 65% found the services helpful.

Despite these unfavorable results, the results show that young people regularly used these services, would generally recommend the services to friends, would use them again, and generally found the services easy to use and satisfactory. Furthermore, young people suggested that these services were accessible and available [51,53,55,71,74], anonymous [51,71,74,75], allowed personal stories to be shared with others [55,73-76], were less stigmatizing than phonelines and face-to-face services [68,79], and trustworthy [53]. This finding suggests that online services fulfil a need, although perhaps do not increase help-seeking. Further exploration into what young people use these services for is warranted.

As with face-to-face services, some barriers to online services remain such as lack of awareness of online resources [51,56,76], young men seeking online help less often than females [68,71,73], some young people’s preference for face-to-face services [73,74], lack of motivation to seek help online [51], the lack of trust of websites [74], and unfavorable content [64,76].

Interestingly, young women were overrepresented in online mental health services in much the same way as in traditional face-to-face care [81]. This could be due to young women using online services, or participating in research, more often than young men. A recent study indicated that young men were generally high use Internet users [82] with 55% seeking help online. Utilizing the advantages of technology by specifically tailoring services for young men may increase rates of young men seeking help online as well as face-to-face [82]. Using the Internet may also assist with young people who prefer face-to-face services by directing young people to appropriate face-to-face services in local areas in the form of online directory services.

Furthermore, online services often include information about mental health literacy [83], providing young people with information about mental illness, where to get help, and what to expect at services. This type of information may increase readiness for care and motivation to seek help.

Another interesting finding was that online services appeared to increase mental health literacy [53,74-77,79], although two studies found no change in mental health literacy [68,78]. Improvements in mental health literacy are likely to assist young people in recognition and management of mental health and may also reduce the self-stigma associated with mental illness [84]. Mental health literacy is associated with seeking help from appropriate treatment and professional services [85]. A recent meta-analysis demonstrated that interventions with a focus on mental health literacy significantly improved help-seeking intentions, although no effect was found for help-seeking behaviors [86]. Ensuring that websites maintain young people’s confidentiality and anonymity appears to be critical to increasing usage of online services.

This systematic review highlights the need for rigorous evaluation methods of online help-seeking programs. The methodology was generally poor across the studies with a high risk of bias. Samples were generally small, the studies often included short-term, if any, follow-up, and non-validated measures of help-seeking behavior and intentions were often used, in some cases with just one question to assess the complex concept of help-seeking. Help-seeking was not the primary outcome for most studies. It is also important to note that help-seeking was not the primary purpose of some of the services, such as discussion forums and self-help programs, where the primary purpose may have been self-help rather than assisting young people to seek help from other services. These methodological issues may account for the lack of change in help-seeking. Also, despite the general satisfaction with online services reported, evaluation of satisfaction was poor. No standard measures of satisfaction were used in the studies limiting the ability to compare user experiences across studies. Short user satisfaction measures are available such as the validated Client Satisfaction Questionnaire (CSQ-3 or 8) [87], which summed together gives an understanding of client’s satisfaction of the service.

Structured online treatment programs, though effective at reducing adolescent depression and anxiety [36-40,88], have poor uptake [89], high dropout rates, and the reduction of mental health symptomatology is not maintained long term [90]. In contrast, unstructured mental health websites have high uptake [77], allowing users to explore the contents, select links that appeal to them, and disregard information that is not relevant or interesting. The primary aim of these services is to give young people information about mental health as well as facilitate help-seeking and pathways to mental health care; however, these aims are rarely evaluated [45]. It is time now to focus on whether these sites facilitate help-seeking and improve well-being to ensure that the online services we provide to young people assist their help-seeking journey and lead to better outcomes and better access to care.

High-quality randomized control trials are needed before the implementation of new services as well as ongoing longitudinal trials to ensure the efficacy of existing services. These trials should include large representative samples, long-term follow-up measures of at least 6-12 months, the use of appropriate, validated measures of help-seeking behavior, help-seeking intentions, beliefs about help-seeking, and client satisfaction. Analyses should also be appropriate and indicate an effect size for future inclusion into meta-analyses.

### Strengths and Limitations

This review is timely and highlights the need to properly evaluate websites aiming to assist young people with their mental health problems and seek help. Including uncontrolled studies in this review allowed for a broad overview of research in this area to date, and as only three RCTs were found, a meta-analysis was not possible. Some studies included were primarily self-help websites, therefore one would hope that further help-seeking was reduced rather than increased. Nevertheless, as the studies themselves included help-seeking as a primary or secondary aim, it can be assumed that help-seeking was considered a goal of these studies. One of the strengths of this review was the focus on both help-seeking behaviors and intentions, as intentions do not always translate into behavior. Further research is needed to explore the mechanisms that facilitate and hinder this process.

### Conclusions

At present, there is a paucity of research exploring the relationship between online services and help-seeking behavior. This is not to say that there is no benefit in online services, rather, that this field has yet to be properly evaluated. Only 35% of young people experiencing mental health problems seek professional face-to-face help [91,92]. Online mental health services may conceivably assist in all elements of the help-seeking process; however, further research into the effectiveness of online services, how they interact with face-to-face services, and whether online services can overcome barriers to mental health care, facilitate readiness for care, and increase help-seeking behavior is needed.

## Multimedia Appendix 1

Terms used for the literature search.

## Multimedia Appendix 2

The adapted Quality Rating Scale.

## Abbreviations

AHSQ: Actual Help Seeking Questionnaire
CIS: Columbia Impairment Scale
QRS: quality rating scale
RCT: randomized controlled trial
