# Supplementary material for: Do Online Mental Health Services Improve Help-Seeking for Young People? A Systematic Review
Source: J Med Internet Res. 2014 Mar 4;16(3):e66. doi: 10.2196/jmir.3103 (PMC3961801; doi:10.2196/jmir.3103)
Supplement: Supplementary file 1 [file jmir_v16i3e66_app1.pdf]

Appendix I. Terms used for the Literature Search

Review publications[1-8] in this area were examined in order to generate lists of key search terms. PsycInfo and MeSH terms were also used.

SEARCH 1: PsycINFO (conducted 27/02/13)

Keyword and subject headings were used in this search as indicated by [K] or [S] respectively. The search was limited by peer-review and English language only.

1. Help seek$[K] OR Seek help$[K] OR Seek treat$[K] OR Help Seeking Behaviour[S] OR Health Care Seeking Behaviour[S] OR Self-referral[S] OR Self Help Techniques[S]

AND

2. Web-base$[K] OR Online[K] OR e-health[K] OR Computer Applications[S] OR Mobile Devices[S] OR Internet[S] OR Information Technology[S]

AND

3. Mental Health[S] OR Mental Disorder[S] OR Mental[K] OR Depress$[K] OR Anxiety[K]

SEARCH 2 & 3: PubMed and the Cochrane Review (conducted 27/02/13)

MeSH terms and “Body - All words” were searched as indicated by [M] or [W] respectively. No limits were set in this search.

1. Diagnostic Self Evaluation[M] OR Self Assessment[M] OR Information Seeking Behaviour[M] OR Help Seek$[W] OR Seek help$[W] OR Self refer$[W]

AND

2. Internet[M] OR Cellular Phone[M] OR Medical Informatics[M] OR Software[M] OR Web Base$[W] OR e-mental health[W] OR Information Technology[W] OR Website[W] OR Online[W] OR Computer Application[W]

AND

3. Mental Health[M] OR Mental Disorders[M] OR Mental[W] OR Depress$[W] OR Anxiety[W]

1. Ahmead M, Bower P. The effectiveness of self help technologies for emotional problems in adolescents: a systematic review. Child and Adolescent Psychiatry and Mental Health [serial on the Internet]. 2008, (1): URL: http://onlinelibrary.wiley.com/o/cochrane/cldare/articles/DARE-12009103512/frame.html. Date accessed:

2. Christensen H, Hickie IB. E-mental health: a new era in delivery of mental health services. *Medical Journal of Australia* 2010;192:S2-S3.

3. Andersson G, Bergstrom J, Buhrman M, Carlbring P, Hollondare F, Kaldo V, Nilsson-Ihrfelt E, Paxling B, Strom L, Waara J. Development of a new approach to guided self-help via the Internet: The Swedish experience. *Journal of Technology in Human Services* 2008;26(2-4):161-81.

4. Barak A, Hen L, Boniel-Nissim M, Shapira Na. A comprehensive review and a meta-analysis of the effectiveness of internet-based psychotherapeutic interventions. *Journal of Technology in Human Services* 2008;26(2-4):109-60.

5. Cavanagh K, Shapiro DA. Computer treatment for common mental health problems. *Journal of clinical psychology* 2004;60(3):239-51. PMID: 14981789.

6. Mair FS, May C, O'Donnell C, Finch T, Sullivan F, Murray E. Factors that promote or inhibit the implementation of e-health systems: an explanatory systematic review. *Bulletin of the World Health Organization* 2012;90(5):357-64. PMID: 22589569.

7. Ybarra ML, Eaton WW. Internet-based mental health interventions. *Mental health services research*

2005;7(2):75-87. PMID: 15974154.
8. Gray NJ, Sesselberg TS, Cantrill JA. Health information-seeking behaviour in adolescence: the place of the internet. *Social science & medicine* 2005;60:1467-78. PMID: 15652680.
