# Supplementary material for: Do Online Mental Health Services Improve Help-Seeking for Young People? A Systematic Review
Source: J Med Internet Res. 2014 Mar 4;16(3):e66. doi: 10.2196/jmir.3103 (PMC3961801; doi:10.2196/jmir.3103)
Supplement: Supplementary file 2 [file jmir_v16i3e66_app2.pdf]

Appendix II. The Adapted Quality Rating Scale

The QRS is a 23-item scale designed to assess the quality of controlled clinical trials for depression and neurosis. As studies that do not involve randomisation and control groups will not be excluded in this review (i.e., qualitative and cross-sectional studies), 4 items ('blinding of subjects', ' blinding of assessor', 'details of side effects' and 'information on comparability') were not applicable and were excluded[41]. An additional item was also added to assess whether the studies included a list of limitation of the study and was rated 0 "No limitations mentioned" 1 "Some major limitations missing" and 2 "Comprehensive list of limitations". These changes resulted in a 20-item scale with each item rated from 0 to 2 and a total range from 0 to 40.

Criteria	Score and rating
**1. Objectives and specification main outcomes a priori**	0 = Objectives unclear<br>1 = Objectives clear but main outcome not specified a priori<br>2 = Objectives clear with a priori specification of main method for assessment of outcome
**2. Adequate sample size**	0 = Less than 50<br>1 = 50 to 100<br>2 = Over 100
**3. Appropriate duration of trial**	0 = No follow-up or less than 3 months<br>1= 3 to 6 months<br>2 = Over 6 months
**4. Power calculation for sample (or appropriate reason for sample size for qualitative/cross-sectional)**	0 = Not reported<br>1 = Mentioned but no details<br>2 = Details of calculation or reason provided
**5. Method of allocation**	0 = Unrandomised and likely to be biased<br>1 = Partially or quasi-randomised with some bias possible<br>2 = Randomised allocation

**6. Concealment of allocation with description of treatments**	0 = Not done or not reported<br>2 = Concealment of allocation code detailed
**7. Clear description of treatments and adjunctive treatment**	0 = Main treatment not clearly described<br>1 = Inadequate details of main or adjunctive treatments<br>2 = Full details of main and adjunctive treatments
**8. Source of subjects described and representative**	0 = Source of subjects not described<br>1 = Source of subjects described but no information on sampling or use of unrepresentative sample (e.g., self-selected)<br>2 = Source of subjects described and representative sample taken (e.g., consecutive admissions, random sample)
**9. Use of diagnostic criteria (or clear specification of inclusion criteria)**	0 = None<br>1= Diagnostic criteria or Clear inclusion criteria<br>2 = Diagnostic criteria and specification of severity
**10. Record of exclusion criteria and number of exclusions and refusals reported**	0 = Criteria and number not reported<br>1 = Criteria or number of exclusions and refusals not reported<br>2 = Criteria and number of exclusions and refusals reported
**11. Description of sample demographics**	0 = Little/ no information (only age/sex)<br>1 = Basic details (e.g., martial status/ethnicity)<br>2 = Full description (e.g., clinical history, socioeconomic status)
**12. Assessment of compliance with experimental treatments (including attendance for therapy)**	0 = Not assessed<br>1 = Assessed for some experimental treatments<br>2 = Assessed for all treatments
**13. Record of number and reasons of withdrawals**	0 = No information on withdrawals by group<br>1 = Withdrawals by group reported without reason<br>2 = Withdrawals and reason by group
**14. Outcome measures described clearly (and**	0 = Main outcomes not clearly described

**therefore replicable) or use of validated (or referenced) instruments**	1 = Some main outcomes not clearly described<br>2 = Main outcomes clearly described or valid and reliable instruments used
**15. ‘Intention to treat’ analysis (all subjects included)**	0 = Less than 95% of subjects included<br>2 = 95% or more included
**16. Presentation of results with inclusion of data for re-analysis of main outcomes (for example, SDs)**	0 = Little data presented<br>1 = Adequate summary statistics or data presented<br>2 = Comprehensive
**17. Appropriate statistical analysis (including correction for multiple tests where applicable)**	0 = Inadequate<br>1 = Adequate<br>2 = Comprehensive and appropriate
**18. Conclusions justified**	0 = No<br>1 = Partially<br>2 = Yes
**19. Declarations of interests (for example, acknowledgements, funding)**	0 = No<br>2 = Yes
**20. Limitations listed**	0 = No limitations listed<br>1 = Some major limitations missing<br>2 = Comprehensive list of limitations
